# Illness Representations of HIV Positive Patients Are Associated with Virologic Success

**DOI:** 10.3389/fpsyg.2016.01991

**Published:** 2016-12-23

**Authors:** Daniela Leone, Lidia Borghi, Giulia Lamiani, Luca Barlascini, Teresa Bini, Antonella d’Arminio Monforte, Elena Vegni

**Affiliations:** ^1^Unit of Clinical Psychology, Department of Health Sciences, University of MilanMilan, Italy; ^2^Department of Biomedical Sciences, Humanitas UniversityMilan, Italy; ^3^Unit of Clinical Psychology, ASST Santi Paolo e Carlo, Presidio San Paolo University HospitalMilan, Italy; ^4^Clinic of Infectious Diseases, Department of Health Sciences, ASST Santi Paolo e Carlo, Presidio San Paolo University Hospital, University of MilanMilan, Italy

**Keywords:** adherence, HAART, HIV, illness representations, IPQ-R, patient engagement, virologic success

## Abstract

**Introduction:** It is important for HIV positive patients to be engaged in their care and be adherent to treatment in order to reduce disease progression and mortality. Studies found that illness representations influence adherence through the mediating role of coping behaviors. However, no study has ever tested if patient engagement to the visits mediate the relationship between illness perceptions and adherence. This study aimed to explore illness representations of HIV positive patients and test the hypothesis that illness representations predict adherence through the mediating role of a component of behavioral engagement.

**Methods:** HIV-positive patients treated with highly active antiretroviral therapy (HAART) for at least one year and presenting to a check-up visit were eligible to participate in the study. Patients completed the Illness Perception Questionnaire-Revised. Behavioral engagement was measured based on the patients’ clinical attendance to the check-up visits; adherence to HAART was measured by viral load. Undetectable viral load or HIV-RNA < 40 copies/ml were considered indexes of virologic success.

**Results:** A total of 161 patients participated in the study. Most of them coherently attributed the experienced symptoms to HIV/HAART; perceived their condition as chronic, stable, coherent, judged the therapy as effective, and attributed their disease to the HIV virus and to their behavior or bad luck. The majority of patients (80.1%) regularly attended check-up visits and 88.5% of them reached virologic success. The mediation model did not show good fit indexes. However, a significant direct effect of two independent variables on virologic success was found. Specifically, the perception that the disease does not have serious consequences on patient’s life and the prevalence of negative emotions toward HIV were associated with virologic success. On the contrary, the patient’s perception that the disease has serious consequences on his/her life and the prevalence of positive emotions were associated with virologic failure. This model showed good fit indexes (CFI = 1; TLI = 1; RMSEA = 0.00; and WRMSR = 0.309).

**Discussion:** Results do not support the mediating role of behavioral engagement in the relationship between illness representations and adherence. As perception of serious consequences coupled with positive emotions are directly associated with virologic failure, clinicians should take them into account to promote treatment adherence.

## Introduction

In the last 20 years, the care of HIV positive patients has changed dramatically. The introduction of highly active antiretroviral therapy (HAART) has allowed patients to achieve an undetectable plasma viral load (HIV – RNA level) and so a virologic success (Das et al., 2010; Gill et al., 2010), thus reducing mortality and morbidity (Mocroft et al., 2003). Due to this pharmacological success, HIV infection has moved from a mortal disease to a chronic condition. The key to virologic success seems to be the adherence to HAART (Gardner et al., 2011). Although viral suppression has become possible with moderate adherence (less than 95%) to HAART regimens, adherence is fundamental in reducing HIV related symptoms, mortality, and the side effects related to the treatments (Paterson et al., 2000; García et al., 2002; Bangsberg, 2006).

It is known that adherence is a complex behavior, influenced by the type of treatment regimen, patient–provider relationship and patient characteristics, including psychological factors (Chesney, 2003; Mills et al., 2006). In order to improve adherence and promote treatment success it is crucial to develop models that predict adherence (DiMatteo et al., 2012) and identify risk factors for non-adherence (Paterson et al., 2000; Ickovics and Meade, 2002; Wood et al., 2003). Psychological models that posit a relation between psychological factors and the management of a chronic illness can be broadly categorized (Horne and Weinman, 1998) as social cognitive models, such as the health belief model (Rosenstock, 1974), stage models, such as the precaution adoption process model (Weinstein, 1988), and the hybrid common-sense self-regulatory model (CS-SRM) of illness representations (Leventhal et al., 1980). The CS-SRM has been adopted by several researchers to predict adherence in several patient populations (e.g., Meyer et al., 1985; Weinman et al., 1996). This model suggests that patients create mental representations of their illness experience through cognitive and emotional processes in order to make sense of it (Leventhal et al., 1997; Cameron and Leventhal, 2003). Cognitive illness representations are composed of five essential dimensions: (1) identity (the nature of the illness and the symptoms the patient considers associated to the disease), (2) cause (personal beliefs about the cause of the illness), (3) timeline (the perceived chronicity of the illness), (4) consequences (perceptions about the short- and long-term effects of the illness), and (5) control (beliefs about the degree of illness and if treatment can be controlled). Beside these cognitive representations, emotions elicited by illness, such as fear, anger, or anxiety, are also integral to illness representations and develop simultaneously with the cognitive components (Leventhal et al., 1997). Based on the CS-SRM, Weinman et al. (1996) developed the Illness Perception Questionnaire (IPQ), in order to measure cognitive illness representations. The IPQ was later revised by Moss-Morris et al. (2002) into the Illness Perception Questionnaire Revised (IPQ-R) to include also the emotional components of patients’ representations.

According to the CS-SRM, when faced with a health-related problem, patients undergo a process, named the self-regulation process, which encompasses three phases: (1) illness representations; (2) coping strategies; and (3) evaluation (Broadbent et al., 2006). First, the patient seeks to understand the illness. From the various internal stimuli, such as illness activity or side effects of the disease, and external information, such as relationship with healthcare providers or public opinion, the cognitive and emotional representations of the health threat are constructed. Thus, illness representations are not necessarily scientifically based as they are often formulated from personal experience, social influences, and/or interaction with healthcare providers. Second, illness representations will lead to the selection of coping strategies to eliminate or control the threat posed by the disease (Broadbent et al., 2006). There are several classifications of coping strategies. One of the most common classifications divided the coping strategies into problem-focused strategies, such as gathering information, and taking action to manage the problem, and emotion-focused strategies aimed to manage distress by minimizing, reducing, or preventing the emotional components of a stressor, such as venting emotions, distracting or using avoidance strategies (Lazarus and Folkman, 1984). Third, patients evaluate the effectiveness of the coping strategy on the outcome or goal of the disease, as, for example, virologic success.

The usefulness and validity of the CS-SRM model has been confirmed by numerous studies on patients suffering from several chronic diseases, such as diabetes, cardiovascular disease, asthma, and cancer (Petrie and Weinman, 1997; Cameron and Leventhal, 2003; French et al., 2013; Mickevičienë et al., 2013). Several studies on different chronic illnesses have confirmed the mediating role of coping strategies in the relationship between illness representations and outcomes, such as mood, quality of life, and patients’ satisfaction (Diefenebach and Leventhal, 1996; Leventhal et al., 2001; Rutter and Rutter, 2002; Hagger and Orbell, 2003; Llewellyn et al., 2007; Gould et al., 2010; Catunda et al., 2016). However, some studies have suggested that illness representations are also directly associated with outcomes regardless of coping strategies, and that illness representations are more strongly associated with outcomes than coping strategies (e.g., Moss-Morris et al., 1996; Scharloo et al., 1998). In addition, some studies that did not measure coping in their research designs have shown strong direct relationships between some illness representations and outcome measures (Petrie et al., 1996; Schiaffino et al., 1998). Illness representations were found to predict patients’ self-management of their disease (Leventhal et al., 1997; Petrie and Weinman, 1997; Cameron and Leventhal, 2003), decisions to seek health care and comply with medical advice (Leventhal et al., 1980), functional adaption and adherence (Moss-Morris et al., 2002).

Overall, these studies highlight that coping strategies may not be the only mediator in the relationship between illness representations and adherence. Recently, patient engagement has received increased attention in the healthcare literature as a psychological factor that may influence adherence. The increasing number of people living with a chronic condition brings to light the importance of engaging patients in their health, helping them to integrate the disease into their identity and life, self-manage their disease and properly use the healthcare resources (Graffigna et al., 2014). A recent model developed by Graffigna et al. (2014) defined patient engagement as a dynamic process, in which patients experience four phases (blackout, arousal, adhesion, and eudaimonic project), each encompassing emotional, cognitive, and behavioral dimensions. According to this model, engagement is the final outcome of a series of emotional, cognitive, and behavioral reframing of the patient’s health condition (Barello and Graffigna, 2014). More specifically, fully engaged patients are able to integrate the disease into their identity and life, manage their own care and mobilize healthcare services proactively if needed. Among the different aspects of engagement, a behavioral dimension is highlighted that relates to the patient adhesion to medical prescriptions, retention in care and visit attendance. The importance of engagement in HIV care has been acknowledged by several authors as it was found to be associated with HIV outcomes and reduced risk transmission behaviors (Marks et al., 2005; Giordano et al., 2007; Metsch et al., 2008; Yehia et al., 2014).

Illness representations and engagement seem to play an important role in understanding HIV positive patients’ self-care behaviors and treatment adherence (Sacajiu et al., 2007; McGavock and Treharne, 2011). However, to our knowledge, no study has ever explored the relationship between illness representations, patient engagement and adherence.

Few studies have explored the relationship between illness representation and adherence in HIV positive patients (Cooper et al., 2009; Reynolds et al., 2009; Pala Norcini and Steca, 2015). Reynolds et al. (2009) found that cognitive illness representations were associated with self-care frequency and effectiveness in the context of HIV care. Cooper et al. (2009) found that adherence to HAART was influenced by individuals’ experiences of both HIV and HAART-related symptoms. Pala Norcini and Steca (2015) identified three configurations of perceptions of illness influence (low, moderate, and high) using the Brief IPQ-R questionnaire. They found that a higher perception of illness influence on patients’ lives (in terms of consequences, negative emotions and intensity of symptoms) was associated with greater viral load, with the mediating role of dysfunctional coping strategies in response to HIV-related stressors. In particular, this research found that high and moderate illness influence perception correlated with passive coping, which consisted in a lack of action in response to HIV-related stressors. Passive coping might also be seen as patients’ perception of helplessness or behavioral disengagement due to their expectation of poor outcomes (Carver et al., 1989).

Based on the results of the literature presented, we hypothesized that the relationship between illness representations and adherence in HIV positive patients could be mediated by behavioral engagement. The present study has two aims: (1) to explore illness representations of HIV positive patients in HAART and (2) to test the hypothesis that illness representations predict adherence through the mediating role of a component of behavioral engagement. Specifically, we tested if regular visit attendance mediates the relationships between illness representations and virologic success.

## Materials and Methods

### Participants

Participants were recruited at the outpatient clinic of infectious diseases of a university hospital in the north of Italy. This center cares for an average of 1000 HIV positive patients per year.

Inclusion criteria were: (1) HIV patients under treatment for at least 1 year; (2) age ≥ 18 years; (3) able to understand and provide informed consent; (4) able to understand Italian according to (depending on) the physician’s and/or researcher’s judgment; (5) no history of psychiatric symptoms; and (6) no actual alcohol or drugs abuse.

### Data Collection

Participants were recruited in the waiting room, before their check-up visit. A researcher with training in clinical psychology presented the study to the patients. Patients who accepted to participated in the study signed an informed consent and were asked to complete the *IPQ-R* (Giardini et al., 2007). Socio-demographic, clinical, and adherence data were collected from inspection of the medical records. The research protocol was approved by the hospital ethics committee.

### Measures

#### Socio-Demographic and Clinical Data

Socio-demographic information that was collected included gender, age, relationship status, educational level, nationality. Clinical information included HIV mode of transmission, year of HIV diagnosis, and therapeutic regimen (fix dose, other drugs, and number of pills per day).

#### Illness Representations

Illness representations were measured with the Italian version of the *IPQ-R* (Giardini et al., 2007). The questionnaire is composed of three sections: (a) identity; (b) opinions, and (c) causes. While items on identity section have dichotomous responses (yes/no), items regarding opinions and causes are rated by patients on a 5-point Likert scale (from “1 = completely disagree” to “5 = completely agree”).

(a) The *identity* section explores patients’ beliefs about the disease’s nature. Out of 14 symptoms, patients are asked to mark the ones they have experienced since being diagnosed with HIV and, which they believed to be linked to the disease/treatment. Two subscales were measured: *reported symptoms* and *associated symptoms*. A high score on identity scales indicates a great number of symptoms experienced and attributed to the disease.

(b) The *opinions* section is composed of 38 items exploring patients’ illness representations. Items are grouped into seven subscales: (1) *timeline* (perception of the disease as chronic); (2) *cyclical symptoms* (perception of a cyclic disease); (3) *consequences* (perception that the disease has serious physical, psychological, and social consequences on the patient’s life); (4) *personal control* (perception that actions can be taken to effectively manage the disease); (5) *treatment control* (high trust in the treatment and its efficacy); (6) *coherence* (high understanding of the disease); and (7) *emotional representations* (prevalence of negative emotions related to the disease).

(c) The *causes* section lists 18 possible illness causes and patients have to rate their level of agreement with each item as a cause of their disease. Only the three most represented causes were used for statistical analysis.

#### Behavioral Engagement

According to Graffigna et al. (2014), engagement is a complex concept encompassing cognitive, emotional, and behavioral dimensions. In this study, a behavioral component of engagement was assessed by measuring the patients’ regular attendance to the last check-up visit. Check-up visits are scheduled by the clinicians every three months, according to the internal guidelines of the clinic of infectious disease where the study was conducted.

#### Adherence

Based on the literature on adherence measures in HIV (Marcellin et al., 2013), an objective index measuring the viral load (level of HIV-RNA copies/ml) was adopted. The use of an objective index is a headway compared to previous studies on predictors of HIV patients’ adherence that commonly used self-reported measures (Ubbiali et al., 2008). An undetectable viral load or HIV-RNA < 40 copies/ml, observed at least at the two previous visits, was considered as virologic success. Virologic success as such is a clinical outcome. However, it was considered as an index of adherence because it is reached only through a regular and correct therapy intake. Indeed, in the clinic of infectious disease where the study was conducted, patients undergo a screening for HIV/RNA baseline, CD4^+^ baseline and drug resistance before starting the HAART treatment. The optimal HAART therapy, which is appropriated also for drug resistance, is then chosen for each patients according to these screening results. Therefore, regardless of their baseline viral load, patients are expected to reach virologic success after three to six months of treatment if they adhere to prescriptions.

### Data Analysis

Means, standard deviations, frequencies, percentages, skewness, and kurtosis were used to describe demographic, clinical, IPQ-R, and adherence data. Subscales with skewness and kurtosis indexes > |1| were normalized with a logarithmic transformation.

Correlations between IPQ-R dimensions (identity and opinions) and socio-demographic variables were conducted. Coefficients were calculated according to the type of socio-demographic variable.

A mediation model was tested through structural equation modeling. IPQ-R dimensions (identity and opinions) were entered as independent variables, clinical attendance as a mediator, and virologic success as dependent variable. The socio-demographic variables which significantly correlated with IPQ-R dimensions were used as control variables. All direct and indirect effects were calculated. The model was evaluated by using the following fit indices: root mean square error of approximation (RMSEA), comparative fit index (CFI), Tucker Lewis index (TLI), and weighted root mean square residual (WRMSR). Values below 0.08 at the RMSEA (Browne and Cudeck, 1993), and values above 0.90 or higher at the CFI and TLI (Bentler, 1990) were judged as indicating an acceptable fit. Values below 0.06 at the RMSEA, and values above 0.95 at the CFI and TLI were judged as indicating a good fit (Hu and Bentler, 1999). We considered values <1 at WRMR as indicating a good fit (Yu, 2002).

The model was modified by removing the variables which did not show significant relations, until a good fit was reached. Data were analyzed using SPSS version 21.0 for Windows and Mplus version 4.0 for Windows.

## Results

Of 231 eligible patients, 171 (74%) participated in the study. Ten patients did not return the questionnaire or returned it incomplete, therefore 161 questionnaires were collected.

Demographic and clinical characteristics of participants are shown in **Table 1**.

**Table 1:** Participants’ socio-demographic and clinical data.

Characteristics	Value
**Gender, *n* (%)**	
Male	120 (74.5)
**Age**	
Mean (*SD*), range	45.2 (10.3), 21–78
**Relationship status, *n* (%)**	
Single	97 (63.8)
Cohabiting/married	39 (25.7)
Divorced/widow	16 (10.5)
**Educational level, *n* (%)**	
Primary	46 (29.3)
High school	83 (52.9)
Graduate	28 (17.8)
**Nationality, *n* (%)**	
Italian	148 (91.9)
Eastern European	1 (0.6)
South American	7 (4.3)
African	3 (1.9)
Asian	2 (1.2)
**HIV mode of transmission, *n* (%)**	
Homosexuals	70 (45.2)
Heterosexuals	48 (31)
Drug addiction	33 (21.3)
Vertical or transfusion transmission	4 (2.6)
**Year of HIV diagnosis**	
Mean (*SD*), range	2000 (8), 1984–2013
**Current fix dose combination regimen, *n* (%)**	
Yes	58 (36)
No	103 (64)
**Other drugs therapies (comorbidities), *n* (%)**	
Yes	76 (47.8)
No	83 (51.6)
**Total number of pills per day**	
Mean (*SD*), range	2.6 (1.8), 1–10

### Illness Perception and Relationship with Socio-Demographic and Clinical Variables

#### Identity

One hundred and forty patients (87%) reported to have suffered from at least one of the symptoms listed since being diagnosed with HIV (mean = 4.8 ± 3.5, range 0–14). The most frequently reported symptoms were: fatigue (67.5%), loss of strength (52.2%), and sleep difficulties (41.3%); 98 patients (60.9%) deemed that at least one of the symptoms experienced was linked to HIV/HAART (mean = 2.6 ± 3.1, range 0–13). The most frequently symptoms associated to HIV/HAART were: loss of strength (72.8%), weight loss (68.5%), and nausea (65%).

#### Opinions

Means illness representation of the seven opinions are reported in **Table 2**. Timeline, personal control, treatment control, and coherence’ means are skewed to the higher values of the IPQ-R range; while cyclical symptoms’ mean is skewed to the lower values of the IPQ-R range.

**Table 2:** Descriptive statistics of participants’ illness representation (IPQ-R opinions).

	Mean *(SD)*	Sample range	IPQ-R opinions range and descriptions
Timeline	23.4 (5)	10–30	Range: 6–30
			High: perception of chronic duration
			Low: perception of acute duration
Cyclical symptoms	9 (3.8)	4–20	Range: 4–20
			High: perception of a cyclic disease
			Low: perception of a stable disease
Consequences	18.4 (3.8)	7–30	Range: 6–30
			High: perception that the disease has serious consequences
			Low: perception that the disease has not serious consequences
Personal control	22.2 (4.2)	6–30	Range: 6–30
			High: perception of high control in disease management
			Low: perception of low control in disease management
Treatment control	20.2 (2.9)	11–25	Range: 5–25
			High: high trust in the disease treatment
			Low: low trust in the disease treatment
Coherence	19.1 (3.9)	5–25	Range: 5–25
			High: high understanding of the disease
			Low: low understanding of the disease
Emotional representations	17.1 (5.5)	6–30	Range: 6–30
			High: prevalence of negative emotions
			Low: prevalence of positive emotions

#### Causes

**FIGURE 1:**
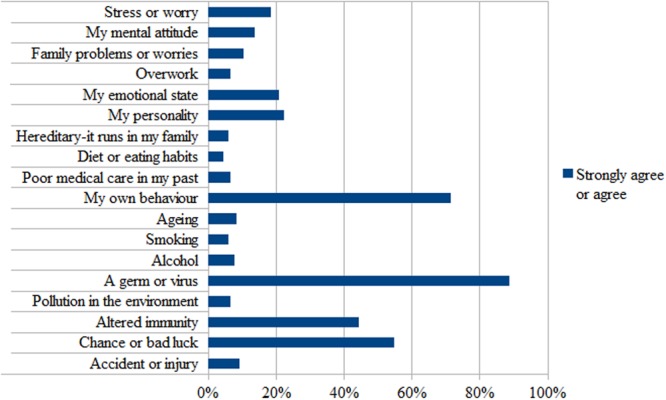
**Participants’ causal attributions for acquiring HIV**.

The three most frequently perceived reasons for becoming HIV were: a germ or virus (88.6%), my own behavior (70.4%), and chance or bad luck (54.1%) (**Figure 1**).

### Relationship between Illness Representation, Behavioral Engagement, and Adherence

The majority of patients (80.1%) showed a regular attendance to the check-up visits, and 88.5% of the patients reached a virologic success. The correlation matrix between IPQ-R dimensions (identity and opinions) and socio-demographic variables is reported in **Table 3**. As gender and educational level correlated with at least one of the study variables, they were included as control variables in the mediation model.

**Table 3:** Correlation matrix between IPQ-R dimensions (identity and opinions) and socio-demographic variables.

	Age^1^	Educational level^2^	Gender^3^	Relationship status ^4^	Year of HIV diagnosis^1^
Timeline	-0.029	0.068	0.052	0.044	-0.021
Cyclical symptoms	0.001	-0.143	0.088	0.156	0.096
Consequences	-0.109	-0.009	0.024	0.146	0.042
Personal control	-0.083	0.135	0.158^∗^	0.168	-0.117
Treatment control	0.045	-0.035	-0.029	0.022	-0.033
Coherence	-0.096	0.071	-0.138	0.151	-0.013
Emotional representations	-0.091	-0.057	0.078	0.115	0.034
Identity reported symptoms	0.045	-0.231^∗∗^	0.187^∗^	0.093	0.131
Identity HIV/HAART associated symptoms	-0.004	-0.108	0.071	0.140	0.129

*^1^Pearson coefficient *r*; ^2^Spearman rho coefficient; ^3^Point-biserial coefficient; ^4^Eta coefficient. ^∗^*p* < 0.05; ^∗∗^*p* < 0.01.*

**FIGURE 2:**
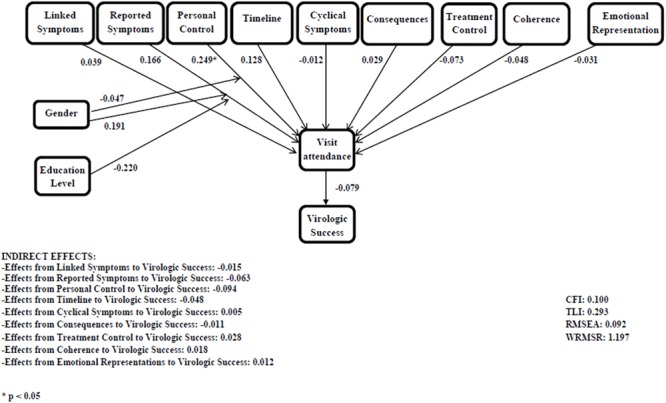
**Mediation model between illness representations, visit attendance, and virologic success**.

**FIGURE 3:**
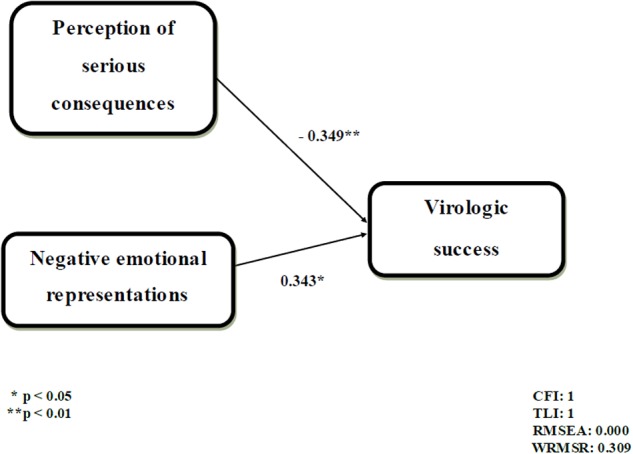
**The path-analysis model of virologic success predictors**.

The initial mediation model did not show good fit indexes (**Figure 2**). Coefficients are shown in standardized form. Specifically, IPQ-R dimensions were not significantly associated with visit attendance, and visit attendance was not significantly associated with virologic success. Significant indirect effects were not detected. Since the mediation model did not fit the data, we tested a direct model between illness representations and virologic success and we proceeded by removing the variables which did not show significant relations, until a model with good fit indexes was obtained. The final path-analysis model is reported in **Figure 3** and presented good fit indexes (CFI = 1; TLI = 1; RMSEA = 0.00; and WRMSR = 0.309). Coefficients are shown in standardized form. Only two independent variables were significantly associated with virologic success: the consequences scale, as a negative predictor, and the emotional representations scale, as a positive predictor. In other words, the perception that the disease does not have serious consequences on the patient’s life and the prevalence of negative emotions were associated with virologic success.

## Discussion

Literature on chronic disease and HIV showed that patients’ illness representations affect illness experiences, management, and adherence (Moss-Morris et al., 1996; Petrie and Weinman, 1997; Spire et al., 2002; Cameron and Leventhal, 2003; Reynolds et al., 2009). The present study aimed to (1) explore illness representations of HIV positive patients in HAART and (2) test the hypothesis that illness representations predict adherence through the mediating role of a component of behavioral engagement.

For what concerns the first aim, results showed that most participants (87%) experienced various symptoms since HIV diagnosis and linked them to HIV and/or HAART coherently with their clinical condition. Overall, patients perceived HIV as a chronic and stable disease. They presented a good understanding of HIV, a general sense of personal control over the disease management and high trust in the treatment. Around 50% of patients did not perceive HIV as having serious consequences on their life and did not report negative related emotions. These results probably reflect the effectiveness of HAART treatment, which has transformed HIV from a terminal to a chronic disease, allowing patients to develop good disease control (Siegel and Lekas, 2002). With regard to the causal attribution for acquiring HIV, our findings highlighted that patients mainly attributed HIV to an “objective” cause (a germ or virus), consistently with a biomedical interpretation of their disease. The majority of patients seemed to be engaged in terms of their attendance to the check-up visits and to have a good adherence to HAART therapy.

As to the relationship between illness representations, behavioral engagement and adherence, our findings show that patients’ attendance to the check-ups does not mediate the relationship between illness representations and virologic success. However, illness representations were found to be directly associated with virologic success. Our findings highlighted that virologic success is related to a specific combination of cognitive and emotional representations in HIV patients. Notably, patients who perceived HIV as not having serious consequences on their life but reported negative emotions connected to the disease were found to reach virologic success. On the contrary, patients who experienced HIV as having more serious consequences on their life, but reported positive emotions, were found to be less adherent, showing a high viral load. According to the theoretical model of patient health engagement (PHE) (Graffigna et al., 2014), which describes the patient engagement process as a transition across four phases, each encompassing emotional, cognitive and behavioral dimensions, our findings seem to describe patients who are positioned at different phases of their health journey. Patients who failed to reach virologic success are those who perceive HIV as having more serious consequences on their lives but, at the same time, present more positive emotions. These patients are probably in the earlier stages of the PHE model and we could hypothesize that, faced with serious illness consequences, they use maladaptive coping strategies, such as emotional detachment and denial, that lead them to refer a prevalence of positive emotions. These patients may not perceive the importance of taking medications for maintaining good health in the long term. These results are consistent with previous studies on HIV infection and heart conditions, which found that patients perceiving their disease as having serious consequences reported higher viral load (Pala Norcini and Steca, 2015) and lower adherence to exercise therapy (Flora et al., 2015). On the contrary, patients who reached virologic success seem to be at an advanced stage of PHE process (such as adhesion and eudaimonic project) as they referred negative emotions related to HIV instead of denying them and, at a cognitive level, they did not perceive HIV as having too many consequences on their lives. Patients in these stages may present a correct intake of the HAART therapy, and thus are more likely to reach virologic success.

The fact that negative emotions were found to be associated with virologic success appears to be in contradiction with previous literature which identified depressive symptoms as the most consistent predictor of treatment non-adherence in HIV (Paterson et al., 2000; Ickovics and Meade, 2002; Ammassari et al., 2004) and other diseases (DiMatteo et al., 2000). This discrepancy may be due to the conceptual difference between emotional representations and depression. According to the CS-SRM, the emotional representations of illness reflect the patient’s cognitive evaluation of the emotional impact of the illness (Moss-Morris et al., 2002). Emotional representations refer to a cognitive elaboration of the emotional impact of the disease and play an important role in the adaptation process. Emotional representations can indeed motivate the patient to develop an action plan, or can be so overwhelming, resulting in less or no action taken with respect to the disease (Diefenebach and Leventhal, 1996). On the contrary, according to the CS-SRM the depressive symptoms are considered an illness outcome and refer to a dysfunctional emotional reaction that is not necessarily processed by a cognitive elaboration. Future studies should explore the mechanism by which emotional representations are translated into lived emotions, and should investigate the relationship between emotional representation and emotional outcomes (e.g., depression) in relation to adherence to HAART among HIV positive patients.

Interestingly, visit attendance did not mediate the relationship between illness representations and adherence. The lack of mediation may be differently discussed. It is possible that the use of visit attendance as an index of behavioral engagement may have reduced the complexity of the construct of patient engagement yielding non-significant results. It is also possible that attendance to the visits, as a form of problem-focused coping strategy, was not effective in order to reduce viral load among HIV patients. A recent meta-analysis (Dempster et al., 2015) highlighted that different coping strategies may work best for different conditions. It is possible that other problem-focused coping strategies should be considered or that emotional-focused strategies (e.g., acceptance/self-blame, avoidance, or social support) may be more effective to mediate or moderate the relationship between illness representations and virologic success in HIV positive patients.

Overall, our findings provided us with a more comprehensive picture of the relationships between illness representation, attendance to the visits and HAART adherence among HIV positive patients. Significant implications for theory and practice can be highlighted. At a theoretical level, our study has advanced research on adherence to HAART compared to previous literature which has mainly focused on socio-demographic, treatment-related or psychological characteristics without exploring the relationships between variables. As illness representations were not found to be associated with visit attendance, but were found to predict adherence, future research could explore the impact of other variables on visit attendance and virologic success, such as coping strategies. Self-blame or acceptance coping were found to mediate the effect of perceived consequences on outcomes among patients with irritable bowel syndrome. Patients who reported little consequences were more likely to accept the illness, achieve a better quality of life and be more satisfied with their health (Rutter and Rutter, 2002). In addition, the role of perceived social stigma could be assessed in relationship to visit attendance and virologic success.

At a practical level, our results shed light on specific illness representations that lead to virologic failure in HIV positive patients. Perceived consequences and emotional representations are potentially modifiable factors that may be targeted in future interventions to enhance patient adherence to HAART therapy, as already done for other illness and outcomes (Petrie et al., 2002; Llewellyn et al., 2007; Phillips et al., 2012). As reported by Llewellyn et al. (2007) in a study on patients with head and neck cancer, illness representations could be targeted for intervention in the time period between diagnosis and shortly after treatment in order to maximize longitudinal outcomes. Psychological group interventions could be implemented for HIV patients presenting specific illness representations in order to help them cope with the disease and process their emotions, thus promoting illness adaptation and empowerment. Another practical implication concerns also clinician-patient communication. The importance of exploring patients’ illness experience to promote adherence has been mentioned by several authors (Zolnierek and DiMatteo, 2009) and has been embraced by a patient-centered model of care (Borghi et al., 2016). During clinical encounters, clinicians should pay particular attention to their patients’ illness representations regarding HIV as a deeper understanding of the patients’ perspective could promote patient adherence (Borghi et al., 2016).

Our study has several limitations that are principally due to the convenience sample recruited, which presented a high prevalence of adherent patients as well as the small number of patients. Therefore, the generalizability of the findings is limited. Moreover, as the study is cross-sectional, the relationship between illness perception, attendance to visits and viral load have to be interpreted with caution especially for what concerns the direction of causality. Finally, our study focused on illness representations and visit attendance, but did not investigate the role of other variables in affecting adherence in HIV patients.

## Conclusion

Our findings revealed a perception of good personal control in disease management, high trust in the therapy and good adherence to treatment. Since patients’ perceived consequences and emotions influence virologic success, clinicians should explore them in order to promote adherence to treatment.

## Ethics Statement

The research protocol was approved by the San Paolo Hospital Ethics Committee. Patients who accepted to participate in the study signed an informed consent.

## Author Contributions

DL, EV, TB, and AdM contributed to the conception and design of the work; DL contributed to the acquisition of data; LiB and LuB contributed to the data analysis; all authors contributed to the interpretation of data. DL, LiB, GL, and LuB contributed to the draft of the work; EV, TB, and AdM revised the work critically. All authors gave their final approval of the version to be published and agreed to be accountable for all aspects of the work in ensuring that questions related to the accuracy or integrity of any part of the work are appropriately investigated and resolved.

## Conflict of Interest Statement

The authors declare that the research was conducted in the absence of any commercial or financial relationships that could be construed as a potential conflict of interest.
